# MEArec: A Fast and Customizable Testbench Simulator for Ground-truth Extracellular Spiking Activity

**DOI:** 10.1007/s12021-020-09467-7

**Published:** 2020-07-09

**Authors:** Alessio Paolo Buccino, Gaute Tomas Einevoll

**Affiliations:** 1 Centre for Integrative Neuroplasticity (CINPLA), University of Oslo, Oslo, Norway; 2 Present Address: Bio Engineering Laboratory, Department of Biosystems Science and Engineering, ETH Zürich, Zürich, Switzerland; 3 Faculty of Science and Technology, Norwegian University of Life Sciences, Ås, Norway

**Keywords:** Spike sorting testbench, Benchmark data, Extracellular recordings simulator, Open-source software

## Abstract

When recording neural activity from extracellular electrodes, both *in vivo* and *in vitro*, spike sorting is a required and very important processing step that allows for identification of single neurons’ activity. Spike sorting is a complex algorithmic procedure, and in recent years many groups have attempted to tackle this problem, resulting in numerous methods and software packages. However, validation of spike sorting techniques is complicated. It is an inherently unsupervised problem and it is hard to find universal metrics to evaluate performance. Simultaneous recordings that combine extracellular and patch-clamp or juxtacellular techniques can provide ground-truth data to evaluate spike sorting methods. However, their utility is limited by the fact that only a few cells can be measured at the same time. Simulated ground-truth recordings can provide a powerful alternative mean to rank the performance of spike sorters. We present here MEArec, a Python-based software which permits flexible and fast simulation of extracellular recordings. MEArec allows users to generate extracellular signals on various customizable electrode designs and can replicate various problematic aspects for spike sorting, such as bursting, spatio-temporal overlapping events, and drifts. We expect MEArec will provide a common testbench for spike sorting development and evaluation, in which spike sorting developers can rapidly generate and evaluate the performance of their algorithms.

**Electronic supplementary material:**

The online version of this article (10.1007/s12021-020-09467-7) contains supplementary material, which is available to authorized users.

## Introduction

Extracellular neural electrophysiology is one of the most used and important techniques to study brain function. It consists of measuring the electrical activity of neurons from electrodes in the extracellular space, that pick up the electrical activity of surrounding neurons. To communicate with each other, neurons generate action potentials, which can be identified in the recorded signals as fast potential transients called *spikes*.

Since electrodes can record the extracellular activity of several surrounding neurons, a processing step called spike sorting is needed. Historically this has required manual curation of the data, which in addition to being time consuming also introduces human bias to data interpretations. In recent years, several automated spike sorters have been developed to alleviate this problems. Spike sorting algorithms (Rey et al. 2015; Lefebvre et al. 2016) attempt to separate spike trains of different neurons (units) from the extracellular mixture of signals using a variety of different approaches. After a pre-processing step that usually involves high-pass filtering and re-referencing of the signals to reduce noise, some algorithms first detect putative spikes above a detection threshold and then cluster the extracted and aligned waveforms in a lower-dimensional space (Quiroga et al. 2004; Rossant et al. 2016; Chung et al. 2017; Hilgen et al. 2017; Jun et al. 2017). Another approach consists of finding spike templates, using clustering methods, and then matching the templates recursively to the recordings to find when a certain spike has occurred. The general term for these approaches is template-matching (Pachitariu et al. 2016; Yger et al. 2018; Diggelmann et al. 2018). Other approaches have been explored, including the use of independent component analysis (Jäckel et al. 2012; Buccino et al. 2018) and semi-supervised approaches (Lee et al. 2017).

The recent development of high-density silicon probes both for *in vitro* (Berdondini et al. 2009; Frey et al. 2009) and *in vivo* applications (Neto et al. 2016; Jun et al. 2017) poses new challenges for spike sorting (Steinmetz et al. 2018). The high electrode count calls for fully automatic spike sorting algorithms, as the process of manually curating hundreds or thousands of channels becomes more time consuming and less manageable. Therefore, spike sorting algorithms need to be be capable of dealing with a large number of units and dense probes. To address these requirements, the latest developments in spike sorting software have attempted to make algorithms scalable and hardware-accelerated (Pachitariu et al. 2016; Jun et al. 2017; Yger et al. 2018; Pachitariu et al. 2019).

The evaluation of spike sorting performance is also not trivial. Spike sorting is unsupervised by definition, as the recorded signals are only measured extracellularly with no knowledge of the underlying spiking activity. A few attempts to provide ground-truth datasets, for example by combining extracellular and patch-clamp or juxtacellular recordings (Henze et al. 2000; Harris et al. 2000; Neto et al. 2016; Yger et al. 2018; Marques-Smith et al. 2018; Allen et al. 2018) exist, but the main limitation of this approach is that only one or a few cells can be patched at the same time, providing very limited ground-truth information with respect to the number of neurons that can be recorded simultaneously from extracellular probes. An alternative method consists of adding artificial or previously-sorted and well-isolated spikes in the recordings (hybrid method) (Rossant et al. 2016; Wouters et al. 2019). The hybrid approach is convenient as all the characteristics of the underlying recording are kept. However, only a few hybrid units can be added at a time, and this limits the validation capability of this method.

Biophysically detailed simulated data provide a powerful alternative and complementary approach to spike sorting validation (Einevoll et al. 2012). In simulations, recordings can be built from known ground-truth data for all neurons, which allows one to precisely evaluate the performance of spike sorters. Simulators of extracellular activity should be able to replicate important aspects of spiking activity that can be challenging for spike sorting algorithms, including bursting modulation, spatio-temporal overlap of spikes, unit drifts over time, as well as realistic noise models. Moreover, they should allow users to have full control over these features and they should be efficient and fast. While simulated recordings provide ground-truth information of many units at once, it is an open question how realistically they can reproduce real recordings.

In the last years, there have been a few projects aiming to develop neural simulators for benchmarking spike sorting methods (Camuñas-Mesa and Quiroga 2013; Hagen et al. 2015; Mondragón-González and Burguière 2017): Camunas et al. developed NeuroCube (Camuñas-Mesa and Quiroga 2013), a MATLAB-based simulator which combines biophysically detailed cell models and synthetic spike trains to simulate the activity of neurons close to a recording probe, while noise is simulated by the activity of distant neurons. NeuroCube is very easy to use with a simple and intuitive graphical user interface (GUI). The user has direct control of parameters to control the rate of active neurons, their firing rate properties, and the duration of the recordings. The cell models are shipped with the software and recordings can be simulated on a single electrodes or a tetrode. It is relatively fast, but the cell model simulations (using NEURON (Carnevale and Hines 2006)) are re-simulated for every recording.

Hagen et al. developed ViSAPy (Hagen et al. 2015), a Python-based simulator that uses multi-compartment simulation of single neurons to generate spikes, network modeling of point-neurons in NEST (Diesmann and Gewaltig 2001) to generate synaptic inputs onto the spiking neurons, and experimentally fitted noise. ViSAPy runs a full network simulation in NEURON (Carnevale and Hines 2006) and computes the extracellular potentials using LFPy (Lindén et al. 2014; Hagen et al. 2018). ViSAPy implements a Python application programming interface (API) which allows the user to set multiple parameters for the network simulation providing the synaptic input, the probe design, and the noise model generator. Cell models can be freely chosen and loaded using the LFPy package. Further, 1-dimensional drift can be incorporated in the simulations by shifting the electrodes over time (Franke et al. 2010). Learning to use the software and, in particular, tailoring the specific properties of the resulting spike trains, for example burstiness, requires some effort by the user. As the running of NEURON simulations with biophysically detailed neurons can be computationally expensive, the use of ViSAPy to generate long-duration spike-sorting benchmarking data is boosted by access to powerful computers.

Mondragon et al. developed a Neural Benchmark Simulator (NBS) (Mondragón-González and Burguière 2017) extending the NeuroCube software. NBS extends the capability of NeuroCube for using user-specific probes, and it combines the spiking activity signals (from NeuroCube), with low-frequency activity signals, and artifacts libraries shipped with the code. The user can set different weight parameters to assemble the spiking, low-frequency, and artifact signals, but these three signal types are not modifiable.

Despite the existence of such tools for generating benchmarking data, their use in spike sorting literature has until now been limited, making the benchmarking and validation of spike sorting algorithms non-standardized and unsystematic. A natural question to ask is thus how to best stimulate the use of such benchmarking tools in the spike sorting community.

From a spike sorting developer perspective, we argue that an ideal extracellular simulator should be *i)* fast, *ii)* controllable, *iii)* biophysically detailed, and *iv)* easy to use. A fast simulator would enable spike sorter developers to generate a large and varied set of recordings to test their algorithms against and to improve their spike sorting methods. Controllability refers to the possibility to have direct control of features of the simulated recordings. The ideal extracellular spike simulator should include the possibility to use different cell models and types, to decide the firing properties of the neurons, to control the rate of spatio-temporal spike collisions, to generate recordings on different probe models, and to have full reproducibility of the simulated recordings. A biophysically detailed simulator should be capable of reproducing key physiological aspects of the recordings, including, but not limited to, bursting spikes, drifts between the electrodes and the neurons, and realistic noise profiles. Finally, to maximize the ease of use, the ideal extracellular simulator should be designed as an accessible and easy to learn software package. Preferably, the tool should be implemented with a graphical user interface (GUI), a command line interface (CLI), or with a simple application programming interface (API).

With these principles in mind, we present here MEArec, an open-source Python-based simulator. MEArec provides a fast, highly controllable, biophysically detailed, and easy to use framework to generate simulated extracellular recordings. In addition to producing benchmark datasets, we developed MEArec as a powerful tool that can serve as a testbench for optimizing existing and novel spike sorting methods. To facilitate this goal, MEArec allows users to explore how several aspects of recordings affect spike sorting, with full control of challenging features such as bursting activity, drifting, spatio-temporal synchrony, and noise effects, so that spike sorter developers can use it to help their algorithm design.

The source code for MEArec is on Github (https://github.com/alejoe91/MEArec) and the Python package is on PyPi (https://pypi.org/project/MEArec/). An extensive documentation is available (https://mearec.readthedocs.io/), and the code is tested with a continuous integration platform (https://travis-ci.org/). Moreover, all the datsets generated for this article and used to make figures are available on Zenodo (10.5281/zenodo.3696926).

The article is organized as follows: in Section “Getting started with MEArec” we introduce the principles of MEArec and we show how to run simulations with the CLI and Python API. In Section “MEArec features” we explain the different features available in MEArec, including the capability of simulating recordings for MEAs, reproducing bursting behavior, controlling spatio-temporal overlaps, reproducing drifts, and replicating biological noise characteristics. In Section “Testbench for spike sorting development and evaluation” we present the use of MEArec as a testbench for spike sorting development, and its integration with the SpikeInterface framework (Buccino et al. 2019). In Section “Simulation output” we document the simulation outputs and how to save and load them with the MEArec API. Finally, in Section Discussion we discuss the presented software and contextualize it with respect to the state-of-the-art.

## Getting started with MEArec

We start by describing the principle of the MEArec simulator and showing examples on how to get started with the simulations.

**Fig. 1:**
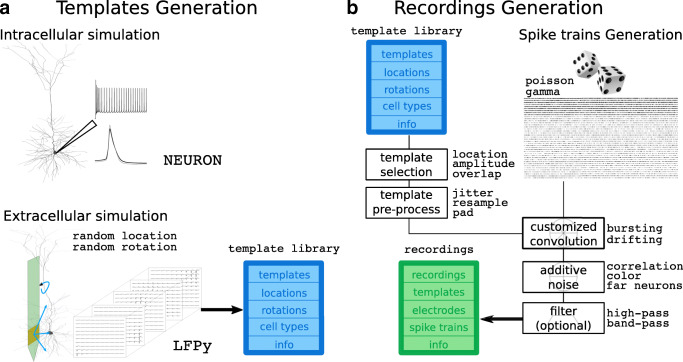
Overview of the MEArec software. The simulation is divided in two phases: *templates generation* and *recordings generation*. **a** The *templates generation* phase is split in an intracellular and extracellular simulation. The intracellular simulation computes, for each available cell model, the transmembrane currents generated by several action potentials. In the extracellular simulation, each cell model is randomly moved and rotated several times and the stored currents are loaded to the model to compute the extracellular action potential, building a *template library*. **b** The *recordings generation* phase combines templates selected from the *template library* and randomly generated spike trains. Selected templates are pre-processed before a customized convolution with the spike trains. Additive noise is added to the output of the convolution, and the recordings can be optionally filtered

The simulation is split in two phases: *templates generation* (Fig. 1a) and *recordings generation* (Fig. 1b). Templates (or extracellular action potentials - EAPs) are generated using biophysically realistic cell models which are positioned in the surroundings of a probe model. The templates generation phase is further divided into an *intracellular* and an *extracellular* simulation. During the intracellular simulation, each cell model is stimulated with a constant current and transmembrane currents of action potentials are computed (using NEURON (Carnevale and Hines 2006)) and stored to disk (the *intracellular* simulation is the most time consuming part and storing its output to disk enables one to run it only once). The extracellular simulation uses the LFPy package (Lindén et al. 2014; Hagen et al. 2018) to compute extracellular potentials generated at the electrodes’ locations using the well-established line-source approximation (see Supplementary Methods – *Templates generation* – for details). In particular, the cell morphology is loaded and shifted to a random position around the probe. Additionally, the user can add different rotations to the models. When the cell model is shifted and rotated, the previously computed and stored transmembrane currents are loaded and the EAP is computed. This step is repeated several times for each cell model, for different positions and rotations. The templates generation phase outputs a library of a large variety of extracellular templates, which can then be used to build the recordings. The templates generation phase is the most time consuming, but the same template library can be used to generate multiple recordings. It is therefore recommended to simulate many more templates than needed by a single recording, so that the same template library can be used to simulate a virtually infinite number of recordings.

MEArec, at installation, comes with 13 layer 5 cortical cell models from the Neocortical Microcircuit Portal (Ramaswamy et al. 2015). This enables the user to dive into simulations without the need to download and compile cell models. On the other hand, the initial set of cell models can be easily extended as outlined in the Supplementary Methods – *Templates generation*.

To generate 30 extracellular spikes (also referred as templates) per cell model recorded on a shank tetrode probe, the user can simply run this command: 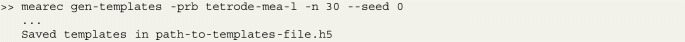


The -prb option allows for choosing the probe model, -n controls the number of templates per cell model to generate, and the --seed option is used to ensure reproducibility and if it is not provided, a random seed is chosen. In both cases, the seed is saved in the HDF5 file, so that the same templates can be replicated.

Recordings are then generated by combining templates selected with user-defined rules (based on minimum distance between neurons, amplitudes, spatial overlaps, and cell-types) and by simulating spike trains (Supplementary Methods – *Recordings generation* – for details on spike trains generation and template selection). Selected templates and spike trains are assembled using a customized (or modulated) convolution, which can replicate interesting features of spiking activity such as bursting and drift. After convolution, additive noise is generated and added to the recordings. Finally, the output recordings can be optionally filtered with a band-pass or a high-pass filter. Note that filtering the recordings will affect the shape and amplitude of the spike waveforms, but this is a common procedure in spike sorting to remove lower frequency components.

**Fig. 2:**
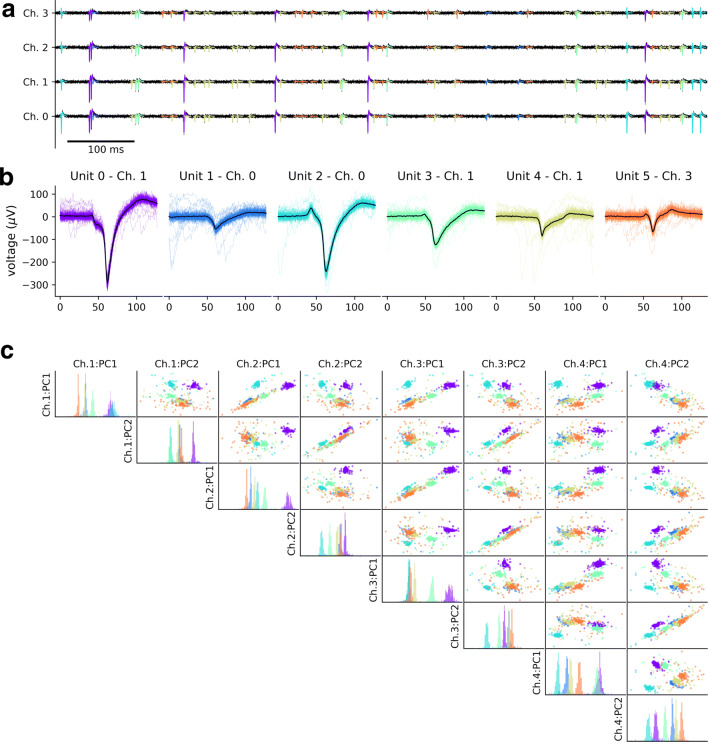
Example of simulated tetrode recording. **a** One second of the recording timeseries on the four tetrode channels. The templates for the different units are overlapped to the recording traces in different colors. **b** Extracted waveforms on the channel with the largest amplitude for the six units in the recordings. **c** PCA projections on the first two PC components of the four tetrode channels. Each color corresponds to a neuron. The diagonal plots display the histograms of the PC projection on the corresponding channel

Recordings can be generated with the CLI as follows: 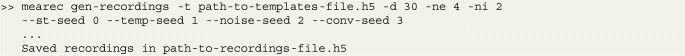


The gen-recordings command combines the selected templates from 4 excitatory cells (-ne 4) and 2 inhibitory cells (-ni 2), that usually have a more narrow spike waveform and a higher firing rate, with randomly generated spike trains. The duration of the output recordings is 30 seconds (-d 30). In this case, four random seeds control the spike train random generation (--st-seed 0), the template selection (--temp-seed 1), the noise generation (--noise-seed 2), and the convolution process (--conv-seed 3). Figure 2 shows one second of the generated recordings (A), the extracted waveforms and the mean waveforms for each unit on the electrode with the largest peak (B), and the principal component analysis (PCA) projections of the waveforms on the tetrode channels.

MEArec also implements a convenient Python API, which is run internally by the CLI commands. For example, the following snippet of code implements the same commands shown above for generating templates and recordings: 
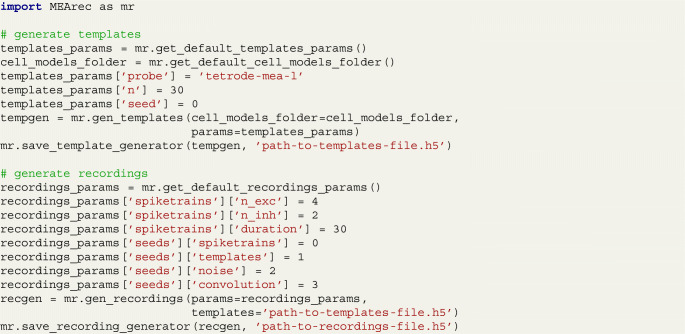


Moreover, the Python API implements plotting functions to visually inspect the simulated templates and recordings. For example, Fig. 2 panels were generated using the plot_recordings() (A), plot_waveforms() (B), and plot_pca_map() (C) functions.

MEArec is designed to allow for full customization, transparency, and reproducibility of the simulated recordings. Parameters for the templates and recordings generation are accessible by the user and documented, so that different aspects of the simulated signals can be finely tuned (see Supplementary Methods for a list of parameters and their explanation). Moreover, the implemented command line interface (CLI) and simple Python API, enable the user to easily modify parameters, customize, and run simulations.

Finally, MEArec permits to manually set several random seeds used by the simulator to make recordings fully reproducible. This feature also enables one to study how separate characteristics of the recordings affect the spike sorting performance. As an example, we will show in the next sections how to simulate a recording sharing all parameters, hence with exactly the same spiking activity, but with different noise levels or drifting velocities.

## MEArec features

### Generation of realistic Multi-Electrode Array recordings

The recent development of Multi-Electrode Arrays (MEAs) enables researchers to record extracellular activity at very high spatio-temporal density both for *in vitro* (Berdondini et al. 2009; Frey et al. 2009) and *in vivo* applications (Neto et al. 2016; Jun et al. 2017). The large number of electrodes and their high density can result in challenges for spike sorting algorithms. It is therefore important to be able to simulate recordings from these kind of neural probes.

To deal with different probe designs, MEArec uses another Python package (MEAutility - https://meautility.readthedocs.io/), that allows users to easily import several available probe models and to define custom probe designs. Among others, MEAutility include Neuropixels probes (Jun et al. 2017), Neuronexus commercial probes (http://neuronexus.com/products/neural-probes/), and a wide variety of square MEA designs with different contact densities (the list of available probes can be found using the mearec available-probes command).

Similarly to the tetrode example, we first have to generate templates for the probes. These are the commands to generate templates and recordings for a Neuropixels design with 128 electrodes (Neuropixels-128). The recordings contain 60 neurons, 48 excitatory and 12 inhibitory. With similar commands, we generated templates and recordings for a Neuronexus probe with 32 channels (A1x32-Poly3-5mm-25s-177-CM32 - Neuronexus-32) with 20 cells (16 excitatory and 4 inhibitory), and a square 10x10 MEA with 15 *μm* inter-electrode-distance (SqMEA-10-15) and 50 cells (40 excitatory and 10 inhibitory). 
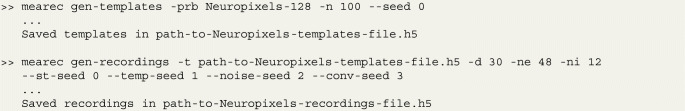


**Fig. 3:**
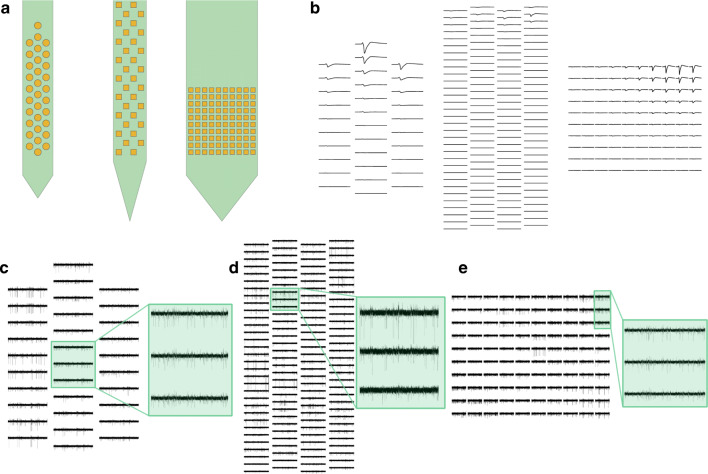
Generation of high-density multi-electrode array recordings. **a** Example of three available probes: a commercial Neuronexus probe (left), the Neuropixels probe (middle), and a high-density square MEA. **b** Sample templates for each probe design. (C-D-E) One-second snippets of recordings from the Neuronexus probe **c**, the Neuropixels probe **d**, and the square MEA probe **e**. The highlighted windows display the activity over three adjacent channels and show how the same spikes are seen on multiple sites

Figure 3 shows the three above-mentioned probes (A), a sample template for each probe design (B), and one-second snippets of the three recordings (C-D-E), with zoomed in windows to highlight spiking activity.

While all the recordings shown so far have been simulated with default parameters, several aspects of the spiking activity are critical for spike sorting. In the next sections, we will show how these features, including bursting, spatio-temporal overlapping spikes, drift, and noise assumptions can be explored with MEArec simulations.

### Bursting modulation of spike amplitude and shape

Bursting activity is one of the most complicated features of spiking activity that can compromise the performance of spike sorting algorithms. When a neuron bursts, i.e., fires a rapid train of action potentials with very short inter-spike intervals, the dynamics underlying the generation of the spikes changes over the bursting period (Hay et al. 2011). While the bursting mechanism has been largely studied with patch-clamp experiments, combined extracellular-juxtacellular recordings (Allen et al. 2018) and computational studies (Hagen et al. 2015) suggest that during bursting, extracellular spikes become lower in amplitude and wider in shape.

In order to simulate this property of the extracellular waveforms in a fast and efficient manner, templates can be modulated both in amplitude and shape during the convolution operation, depending on the spiking history.

**Fig. 4:**
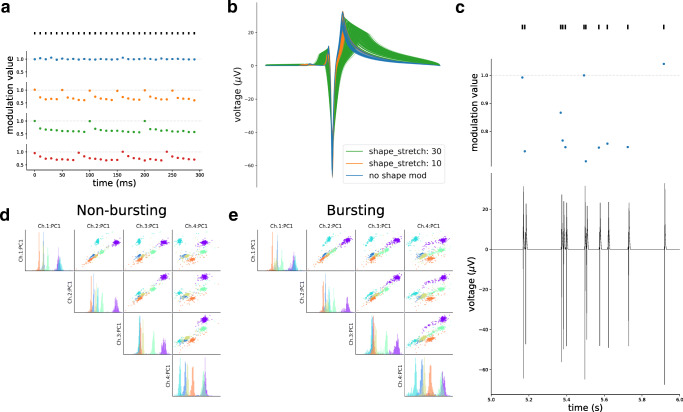
Bursting behavior. **a** Modulation values computation for a sample spike train of 300 *ms* with constant inter-spike-intervals of 10 *ms*. The blue dots show the modulation values for each spike when bursting is not activated: each value is drawn from a $\mathcal {N}(1, 0.05^{2})$ distribution. When bursting is activated, a bursting event can be limited by the maximum number of spikes (orange - 5 spikes, green - 10 spikes), or by the maximum bursting event duration (red - 75 *ms*). **b** Modulated templates. The blue lines show templates modulated in amplitude only. The orange and green lines display the same templates with added shape modulation. **c** Modulation in tetrode recordings. The top panel shows spikes in a one-second period. The middle panel displays the modulation values for those spikes. The bottom panel shows the modulated template on the electrode with the largest peak after convolution. (D-E) PCA projections on the first principal component for the tetrode recordings without bursting (the same as Section Getting started with MEArec) **d** and re-simulated with bursting **e** and shape modulation enabled. Note that the PCA projections were computed in both cases from the waveforms without bursting. The clusters, with bursting, become more spread and harder to separate than without bursting

To demonstrate how bursting is mimicked, we built a toy example with a constant spike train with 10 *ms* inter-spike-interval (Fig. 4a). A *modulation value* is computed for each spike and it is used to modulate the waveform for that event by scaling its amplitude, and optionally stretching its shape. The blue dots show the default modulation (bursting disabled), in which the modulation values are drawn from a Gaussian distribution with unitary mean to add some physiological variation to the spike waveforms. When bursting is enabled (by setting the bursting parameter to true), the modulation values are computed based on the spike history, and it depends on the number of consecutive spikes in a bursting event and their average inter-spike-intervals (see Supplementary Methods – *Recordings generation - Modulated convolution* – for details on the modulation values calculation).

Bursting events can be either controlled by the maximum number of spikes making a burst (orange dots - 5 spikes per burst; green dots - 10 spikes per burst) or by setting a maximum bursting duration (red dots - maximum 75 *ms*). Note that in Fig. 4a the spike train is constant just to illustrate the computation of the modulation values. In actual simulations, instead, the modulation values will depend on the firing rate and the timing between spikes.

By default, spikes are only modulated in amplitude. The user can also enable shape modulation by setting the shape_mod parameter to true. The modulation value, computed for each spike, controls both the amplitude scaling and shape modulation of the spike event. For amplitude modulation, the amplitude of the spike is simply multiplied by the modulation value. Additionally, when shape modulation is enabled, the waveform of each spike is also stretched. The shape_stretch parameter controls the overall amount of stretch, but the actual stretch of single waveforms depends on the modulation value computed for each spike. In Fig. 4b, examples of bursting templates are shown. The blue traces display templates only modulated in amplitude, i.e., the amplitude is scaled by the modulation value. The orange and green traces, instead, also present shape modulation, with different values of the shape_stretch parameter (the higher the shape_stretch, the more stretched waveforms will be). We refer to the Supplementary Methods – *Recordings generation - Modulated convolution* – for further details on amplitude and shape modulation.

Figure 4c shows a one-second snippet of the tetrode recording shown previously after bursting modulation is activated. The top panel shows the spike events, the middle one displays the modulation values computed for each spike, and the bottom panel shows the output of the modulated convolution between one of the templates (on the electrode with the largest amplitude) and the spike train.

Figures 4d and e show the waveform projections on the first principal component of each channel for the tetrode recording shown in Section Getting started with MEArec with and without bursting enabled, respectively. In this case all neurons are bursting units and this causes a stretch in the PCA space, which is a clear complication for spike sorting algorithms. Note that shape modulation does not affect all neurons by the same amount, since it depends on the spike history and therefore on the firing rate.

### Controlling spatio-temporal overlaps

Another complicated aspect of extracellular spiking activity that can influence spike sorting performance is the occurrence of overlapping spikes. While temporal overlapping of events on spatially separated locations can be solved with feature masking (Rossant et al. 2016), spatio-temporal overlapping can cause a distortion of the detected waveform, due to the superposition of separate spikes. Some spike sorting approaches, based on template-matching, are designed to tackle this problem (Pachitariu et al. 2016; Yger et al. 2018; Diggelmann et al. 2018).

**Fig. 5:**
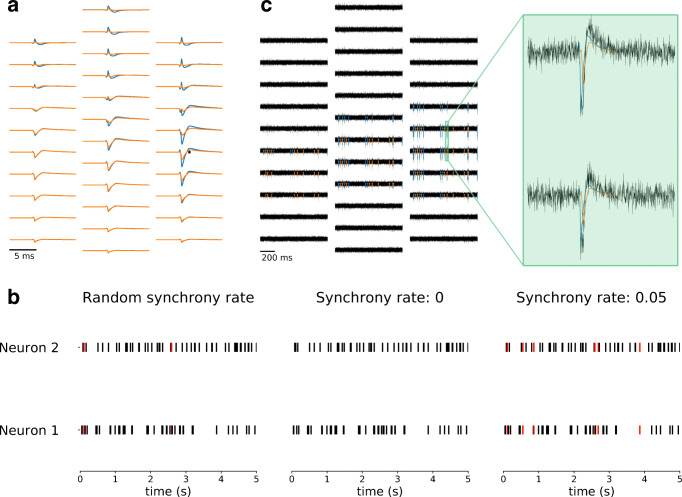
Controlling spatio-temporal overlapping spikes. **a** Example of two spatially overlapping templates. The two templates are spatially overlapping because on the electrode with the largest signal (depicted as an black asterisk) for the blue template, the orange template has an amplitude greater than the 90% of its largest amplitude. **b** Without setting the synchrony rate, the random spike trains (left) present a few spatio-temporal collisions (red events). When setting the synchrony rate to 0 (middle), the spatio-temporal overlaps are removed. When the synchony rate is set to 0.05 (right), spatio-temporal overlapping spikes are added to the spike trains. **c** One-second snippet of the recording with 0.05 synchrony. In the magnified window, a spatio-temporal overlapping event is shown: the collision results in a distortion of the waveform

In order to evaluate to what extent spatio-temporal overlap affects spike sorting, MEArec allows the user to set the number of spatially overlapping templates and to modify the synchrony rate of their spike trains. In Fig. 5 we show an example of this on a Neuronexus-32 probe (see Fig. 3A). The recording was constructed with two excitatory and spatially overlapping neurons, whose templates are shown in Fig. 5a (see Supplementary Methods – *Recordings generation - Overlapping spikes and spatio-temporal synchrony* – for details on the spatial overlap definition). The spike synchrony rate can be controlled with the sync_rate parameter. If this parameter is not set (Fig. 5b - left), some spatio-temporal overlapping spikes are present (red events). If the synchrony rate is set to 0, those spikes are removed from the spike trains (Fig. 5b - middle). If set to 0.05, i.e., 5% of the spikes will be spatio-temporal collisions, events are added to the spike trains to reach the specified synchrony rate value of spatio-temporal overlap. As shown in Fig. 5c, the occurrence of spatio-temporal overlapping events affects the recorded extracellular waveform: the waveforms of the neurons, in fact, get summed and might be mistaken for a separate unit by spike sorting algorithms when the spikes are overlapping.

The possibility of reproducing and controlling this feature of extracellular recordings within MEArec could aid in the development of spike sorters which are robust to spatio-temporal collisions.

### Generating drifting recordings

When extracellular probes are inserted in the brain, especially for acute experiments, the neural tissue might move with respect to the electrodes. This phenomenon is known as drift. Drift can be due to a slow relaxation of the tissue (slow drift) or to fast re-adjustments of the tissue, for example due to an abrupt motion of the tissue (fast drift). These two types of drifts can also be observed in tandem (Pachitariu et al. 2019).

Drifting units are particularly critical for spike sorting, as the waveform shapes change over time due to the relative movement between the neurons and the probe. New spike sorting algorithms have been developed to specifically tackle the drifting problem (e.g. Kilosort2 (Pachitariu et al. 2019), IronClust (Jun et al. 2017)).

**Fig. 6:**
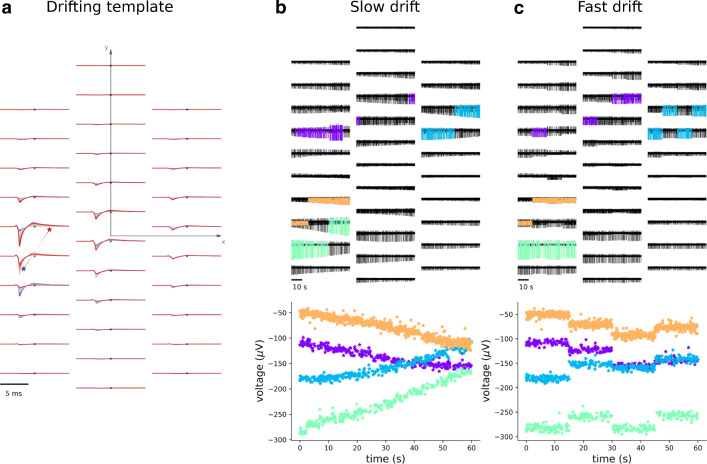
Drifting. **a** Example of a drifting template. The colored asterisks on the left show the trajectory from the initial (blue large asterisk) to the final (red large asterisk) neuron positions. The positions are in the x-y coordinates of the probe plane, and the electrode locations are depicted as black dots. The corresponding templates are displayed at the electrode locations with the same colormap, showing that the template peak is shifted upwards following the soma position. **b** Slow drift. (top) 60-second slow drifting recording with four neurons moving at a velocity of 20 *μ* m/*min*. Templates on the largest electrode are superimposed in different colors on the recordings. Note that the maximum channel changes over time. (bottom) Amplitude of the waveforms over time on the electrode with the largest initial peak. **c** Fast drift. (top) 60-second fast drifting recording with four neurons undergoing a fast drift event every 15 s. Templates on the largest electrode are superimposed in different colors on the recordings. Also for fast drifts, the maximum channel changes over time. (bottom) Amplitude of the waveforms over time on the electrode with the largest initial peak

In order to simulate drift in the recordings, we first need to generate drifting templates: 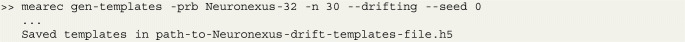


Drifting templates are generated by choosing an initial and final soma position with user-defined rules (see Supplementary Methods – *Template generation - Drifing templates* – for details) and by moving the cell along the line connecting the two positions for a defined number of constant drifting steps that span the segment connecting the initial and final positions (30 steps by default). An example of a drifting template is depicted in Fig. 6a, alongside with the drifting neuron’s soma locations for the different drifting steps.

Once a library of drifting templates is generated, drifting recordings can be simulated. MEArec allows users to simulate recordings with three types of drift modes: *slow*, *fast*, and *slow+fast*. When slow drift is selected, the drifting template is selected over time depending on the initial position and the drifting velocity (5 *μ*/*min* by default). If the final drifting position is reached, the drift direction is reversed. For fast drifts, the position of a drifting neuron is shifted abruptly with a user-defined period (every 20 s by default). The new position is chosen so that the difference in waveform amplitude of the drifting neuron on its current maximum channel remains within user-defined limits (5-20*μV* by default), in order to prevent from moving the neuron too far from its previous position. The slow+fast mode combines the slow and fast mechanisms.

In Fig. 6b and c we show examples of slow drift and fast drift, respectively. In the top panel the recordings are displayed, with superimposed drifting templates on the electrode with the largest peak. Note that the maximum channel can change over time due to drift. In the bottom panels, instead, the amplitude of the waveforms on the channels with the initial largest peak for each neuron are shown over time. Slow drift causes the amplitude to slowly vary, while for fast drifts we observe more abrupt changes when a fast drift event occurs. In the *slow+fast* drift mode, these two effects are combined.

### Modeling experimental noise

Spike sorting performance can be greatly affected by noise in the recordings. Many algorithms first use a spike detection step to identify putative spikes. The threshold for spike detection is usually set depending on the noise standard deviation or median absolute deviation (Quiroga et al. 2004). Clearly, recordings with larger noise levels will result in higher spike detection thresholds, hence making it harder to robustly detect lower amplitude spiking activity. In addition to the noise amplitude, other noise features can affect spike sorting performance: some clustering algorithms, for example, assume that clusters have Gaussian shape, due to the assumption of an additive normal noise to the recordings. Moreover, the noise generated by biological sources can produce spatial correlations in the noise profiles among different channels and it can be modulated in frequency (Camuñas-Mesa and Quiroga 2013; Rey et al. 2015).

**Fig. 7:**
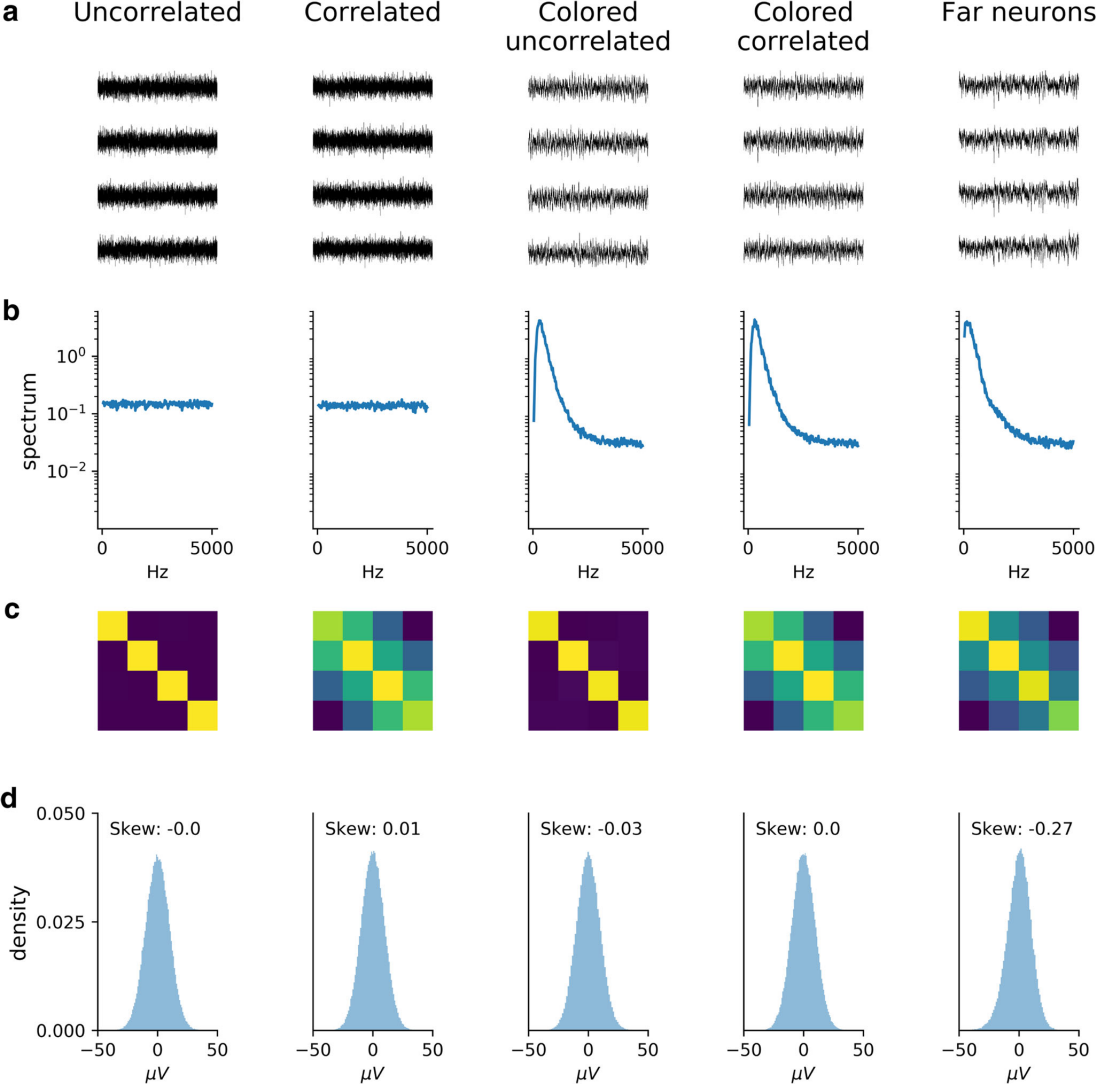
Noise models. The 5 columns refer to different noise models: 1) Uncorrelated Gaussian noise, 2) Distance-correlated Gaussian noise, 3) Colored uncorrelated Gaussian noise, 4) Colored distance-correlated Gaussian noise, and 5) Noise generated by distant neurons. **a** One-second spiking-free recording. **b** Spectrum of the first recording channel between 10 and 5000 Hz. **c** Covariance matrix of the recordings. **d** Distribution of noise amplitudes for the first recording channel. The different noise models vary in the spectrum, channel correlations, and amplitude distributions

To investigate how the above-mentioned assumptions on noise can affect spike sorting performance, MEArec can generate recordings with several noise models. Figure 7 shows 5-second spiking-free recordings of a tetrode probe for five different noise profiles that can be generated (A - recordings, B - spectrum, C - channel covariance, D - amplitude distribution).

The first column shows uncorrelated Gaussian noise, which presents a flat spectrum, a diagonal covariance matrix, and a symmetrical noise amplitude distribution. In the recording in the second column, spatially correlated noise was generated as a multivariate Gaussian noise with a covariance matrix depending on the channel distance. Also in this case, the spectrum (B) presents a flat profile and the amplitude distribution is symmetrical (D), but the covariance matrix shows a correlation depending on the inter-electrode distance. As previous studies showed (Camuñas-Mesa and Quiroga 2013; Rey et al. 2015), the frequency content of extracellular noise is not flat, but its spectrum is affected by the spiking activity of distant neurons, which appear in the recordings as below-threshold *biological* noise. To reproduce the spectrum profile that is observed in experimental data, MEArec allows coloring the noise spectrum of Gaussian noise with a second order infinite impulse response (IIR) filter (see Supplementary Methods – *Recordings generation - Noise models and post-processing* – for details). Colored noise represents an efficient way of obtaining the desired spectrum, as shown in the third and fourth columns of Fig. 7, panel B. Distance correlation is maintained (panel C - fourth column), and the distribution of the noise amplitudes is symmetrical. Finally, a last noise model enables one to generate activity of distant neurons. In this case, noise is built as the convolution between many neurons (300 by default) whose template amplitudes are below an amplitude threshold (10*μV* by default). A Gaussian noise floor is then added to the resulting noise, which is scaled to match the user-defined noise level. The *far-neurons* noise profile is shown in the last column of Fig. 7. While the spectrum and spatial correlation of this noise profile are similar to the ones generated with a colored, distance-correlated noise (4th column), the shape of the noise distribution is skewed towards negative values (panel D), mainly due to the negative contribution of the action potentials.

The capability of MEArec to simulate several noise models enables spike sorter developers to assess how different noise profiles affect their algorithms and to modify their methods to be insensitive to specific noise assumptions.

## Testbench for spike sorting development and evaluation

In the previous sections, we have shown several examples on how MEArec is capable of reproducing several aspects of extracellular recordings which are critical for spike sorting performance, in a fully reproducible way. The proposed design and its integration with a spike sorting evaluation framework called SpikeInterface (Buccino et al. 2019) enables developers to actively include customized simulations in the spike sorting development phase.

**Fig. 8:**
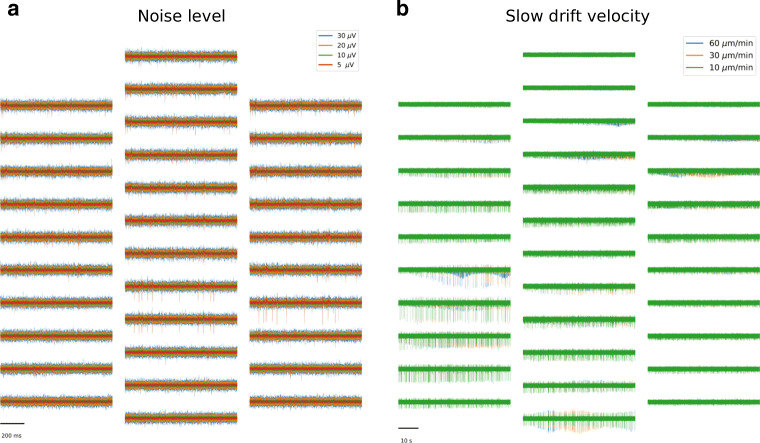
MEArec as testbench platform for spike sorting. **a** Four one-second snippet of recordings generated with a different noise level parameter (5 - red, 10 - green, 20 - orange, and 30*μV* - blue). The underlying spiking activity is exactly the same for all recordings, and the only difference lies in the standard deviation of the underlying uncorrelated Gaussian noise. **b** Three slow drifting recordings generated with a different drifting velocity parameter (10 - green, 30 - orange, and 60 *μ* m/*min* - blue). Also in this case, the underlying spiking activity is the same, but it can be observed how the different speeds result in a modification of waveforms over time

Due to its speed and controllability, we see MEArec as a *testbench*, rather than a *benchmark* tool. We provide here a couple of examples. In Fig. 8a, we show a one-second section of recordings simulated on a Neuronexus-32 probe with fixed parameters and random seeds regarding template selection and spike train generation, but with four different levels of additive Gaussian noise, with standard deviations of 5, 10, 20, and 30 *μV*. The traces show the same underlying spiking activity, so the only variability in spike sorting performance will be due to the varying noise levels. Similarly, in Fig. 8b, 1-minute drifting recordings were simulated with three different drifting velocities. The recordings show that for low drifting speeds the waveform changes are almost not visible (green traces), while for faster drifts (orange and blue traces), the waveform changes over time become more important.

The capability of MEArec of reproducing such behaviors in a highly controlled manner could aid in the design of specific tests for measuring and quantifying the ability of a spike sorting software to deal with specific complexities in extracellular recordings. Other examples include simulating a recording with increasing levels of bursting in order to measure to what extent bursting units are correctly clustered, or changing the synchrony rate of spatially overlapping units to assess how much spatio-temporal collisions affect performance.

### Integration with SpikeInterface

We have recently developed SpikeInterface (Buccino et al. 2019), a Python-based framework for running several spike sorting algorithms, comparing, and validating their results. MEArec can be easily interfaced to SpikeInterface so that simulated recordings can be loaded, spike sorted, and benchmarked with a few lines of code. In the following example, a MEArec recording is loaded, spike sorted with Mountainsort4 (Chung et al. 2017) and Kilosort2 (Pachitariu et al. 2019), and benchmarked with respect to the ground-truth spike times available from the MEArec simulation: 
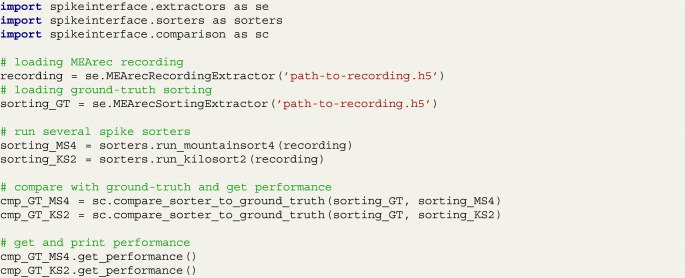


The get_performance() function returns the accuracy, precision, and recall for all the ground-truth units in the MEArec recording. For further details on these metrics and a more extensive characterization of the comparison we refer to the SpikeInterface documentation and article (Buccino et al. 2019).

The combination of MEArec and SpikeInterface represents a powerful tool for systematically testing and comparing spike sorter performances with respect to several complications of extracellular recordings. MEArec simulations, in combination with SpikeInterface, are already being used to benchmark and compare spike sorting algorithms within the SpikeForest project (Magland et al. 2020).

### Performance considerations

As a testbench tool, the speed requirement has been one of the main design principle of MEArec. In order to achieve high speed, most parts of the simulation process are fully parallelized. As shown in Fig. 1, the simulations are split in templates and recordings generation. The templates generation phase is the most time consuming, but the same template library can be used to generate several recordings. This phase is further split in two sub-phases: the intracellular and extracellular simulations. The former only needs to be run once, as it generates a set of cell model-specific spikes that are stored and then used for extracellular simulations, which is instead probe specific.

We present here run times for the different phases of the templates generation and for the recordings generation. All simulations were run on an Ubuntu 18.04 Intel(R) Core(TM) i7-6600U CPU @ 2.60GHz, with 16 GB of RAM.

The intracellular simulation run time for the 13 cell models shipped with the software was $\sim 130$ seconds ($\sim 10$ seconds per cell model).

Run times for extracellular simulations for several probe types, number of templates in the library, and drifting templates are shown in the *Templates generation* section of Table 1. The run times for this phase mainly depend on the number of templates to be generated (N templates column), on the minimum amplitude of accepted templates (Min. amplitude column, see Supplementary Methods – *Templates generation - Extracellular simulation* – for further details), and especially on drift (Drifting column). When simulating drifting templates, in fact, the number of actual extracellular spikes for each cell model is N templates times N drift steps. Note that in order to generate the *far-neurons* noise model, the minimum amplitude should be set to 0, so that low-amplitude templates are not discarded. The number of templates available in the template library will be the specified number of templates (N templates) times the number of cell models (13 by default).

**Table 1:** Templates and recordings generation run times depending on several simulation parameters. The templates simulations were run with 13 concurrent jobs (same as the number of models). The recordings simulations were run with four concurrent jobs, and a chunk duration of 20 s.

Templates generation						
Probe	N templates	N channels	Min. amplitude	Drifting	N drift steps	Run time (s)
tetrode-mea-l	30	4	30	No	-	169
tetrode-mea-l	100	4	30	No	-	588
tetrode-mea-l	100	4	0	No	-	236
Neuronexus-32	100	32	30	No	-	567
Neuropixels-128	100	128	30	No	-	809
SqMEA-10-15	30	100	30	No	-	1027
Neuronexus-32	30	32	30	Yes	50	2000
Recordings generation						
Probe	N cells	N channels	Duration	Bursting	Drifting	Run time (s)
tetrode-mea-l	6	4	10	No	No	0.5
tetrode-mea-l	6	4	600	No	No	7
Neuronexus-32	20	32	30	No	No	8
Neuronexus-32	20	32	30	Yes	No	52
Neuropixels-128	60	128	30	No	No	46
SqMEA-10-15	50	100	30	No	No	33
Neuronexus-32	4	32	60	No	Yes	15
Neuronexus-32	20	32	60	No	Yes	37

Recordings are then generated using the simulated template libraries. In Table 1, the *Recordings generation* section shows run times for several recordings with different probes, durations, number of cells, bursting, and drifting options. The main parameter that affects simulation times is the number of cells, as it increases the number of modulated convolutions. Bursting and drifting behavior also increase the run time of the simulations, because of the extra processing required in the convolution step. The simulation run times, however, range from a few seconds to a few minutes. Therefore, the speed of MEArec enables users to generate numerous recordings with different parameters for testing spike sorter performances. Moreover, the software internally uses memory maps to reduce the RAM usage and the simulations can be *chunked* in time. These features enable users to simulate long recordings on probes with several hundreds of electrodes (e.g. Neuropixels probes) without the need of large-memory nodes or high-performance computing platforms.

## Simulation output

The templates generation outputs a Template Generator object, containing the following fields:

templates: contains the generated templates – array with shape (n_templates, n_electrodes, n_points) for non-drifting templates or (n_templates, n_drift_steps, n_electrodes, n_points) for drifting ones
locations: contains the 3D soma locations of the templates – array with shape (n_templates, 3) for non-drifting templates or (n_templates, n_drift_steps, 3) for drifting templates.
rotations: contains the 3D rotations applied to the cell model before computing the template – array with shape (n_templates, 3) (for drifting templates rotation is fixed)
celltypes: contains the cell types of the generated templates – array of strings with length (n_templates)
info: contains a dictionary with parameters used for the simulation (params key) and information about the probe (electrodes key)

The recordings generation outputs a Recording Generator object, containing the following fields:

recordings: contains the generated recordings – array with shape (n_electrodes, n_samples)
spiketrains: contains the spike trains – list of (n_neurons) neo.Spiketrain objects (Garcia et al. 2014)
templates: contains the selected templates – array with shape (n_neurons, n_jitters, n_electrodes, n_templates samples) templates for non-drifting recordings - or (n_neurons, n_drift_steps, n_jitters, n_electrodes, n_neurons) for drifting ones
templates_celltypes: contains the cell type of the selected templates – array of strings with length (n_neurons)
templates_locations: contains the 3D soma locations of the selected templates – array with shape (n_neurons, 3) for non-drifting recordings or (n_neurons, n_drift_steps, 3) for drifting ones
templates_rotations: contains the 3D rotations applied to the selected templates – array with shape (n_neurons, 3)
channel_positions: contains the 3D positions of the probe electrodes – array with shape (n_electrodes, 3)
timestamps: contains the timestamps in seconds – array with length (n_samples)
voltage_peaks: contains the average voltage peaks of the templates on each electrode – array with shape (n_neurons, n_electrodes)
spike_traces: contains a clean spike trace for each neuron (generated by a clean convolution between the spike train and the template on the electrode with the largest peak) – array with shape (n_neurons, n_samples)
info: contains a dictionary with parameters used for the simulation

When simulating with the Python API, the returned TemplateGenerator and RecordingGenerator can be saved as .h5 files with: 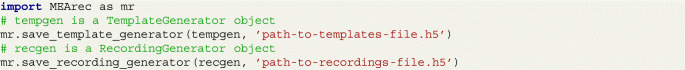


The generation using the CLI saves templates and recordings directly. The saved templates and recordings can be loaded in Python as TemplateGenerator and RecordingGenerator objects with: 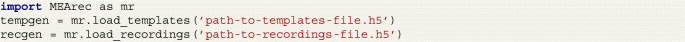


## Discussion

In this paper we have presented MEArec, a Python package for simulating extracellular recordings for spike sorting development and validation. We first introduced an overview of the software function, consisting in separating the templates and the recordings generation to improve efficiency and simulation speed. We then showed the ease of use of the software, whose command line interface and simple Python API enable users to simulate extracellular recordings with a couple of commands or a few lines of code. We explored the capability of reproducing and controlling several aspects of extracellular recordings which can be critical for spike sorting algorithms, including spikes in a burst with varying spike shapes, spatio-temporal overlaps, drifting units, and noise assumptions. We illustrated two examples of using MEArec, in combination with SpikeInterface (Buccino et al. 2019), as a testbench platform for developing spike sorting algorithms. Finally, we benchmarked the speed performance of MEArec (Table 1).

Investigating the validation section of several recently developed spike sorting algorithms (Rossant et al. 2016; Pachitariu et al. 2016; Jun et al. 2017; Hilgen et al. 2017; Jun et al. 2017; Lee et al. 2017; Yger et al. 2018), it is clear that the neuroscientific community needs a standardized validation framework for spike sorting performance. Some spike sorters are validated using a so called hybrid approach, in which well-identified units from previous experimental recordings are artificially injected in the recordings and used to compute performance metrics (Rossant et al. 2016; Pachitariu et al. 2016; Wouters et al. 2019). The use of templates extracted from previously sorted datasets poses some questions regarding the accuracy of the initial sorting, as well as the complexity of the well-identified units. Alternatively, other spike sorters are validated on experimental paired ground-truth recordings (Chung et al. 2017; Yger et al. 2018). While these valuable datasets (Harris et al. 2000; Henze et al. 2000; Neto et al. 2016; Marques-Smith et al. 2018) can certainly provide useful information, the low count of ground-truth units makes the validation incomplete and could result in biases (for example algorithm-specific parameters could be tuned to reach a higher performance for the recorded ground-truth units). A third validation method consist of using simulated ground-truth recordings (Einevoll et al. 2012). While this approach is promising, in combination with experimental paired recordings, the current available simulators (Camuñas-Mesa and Quiroga 2013; Hagen et al. 2015; Mondragón-González and Burguière 2017) present some limitations in terms of biological realism, controllability, speed, and/or ease of use (see Introduction). We therefore introduced MEArec, a software package which is computationally efficient, easy to use, highly controllable, and capable of reproducing critical characteristics of extracellular recordings relevant to spike sorting, including bursting modulation, spatio-temporal overlaps, drift of units over time, and various noise profiles.

The capability of MEArec to replicate complexities in extracellular recordings which are usually either ignored or not controlled in other simulators, permits the user to include tailored simulations in the spike sorting implementation process, using the simulator as a testbench platform for algorithm development. MEArec simulations could not only be used to test the final product, but specific simulations could be used to help implementing algorithms that are able to cope with drifts, bursting, and spatio-temporal overlap, which are regarded as the most complex aspects for spike sorting performance (Rey et al. 2015; Yger et al. 2018).

In MEArec, in order to generate extracellular templates, we used a well-established modeling framework for solving the single neuron dynamics (Carnevale and Hines 2006), and for calculating extracellular fields generated by transmembrane currents (Lindén et al. 2014; Hagen et al. 2015). These models have some assumptions that, if warranted, could be addressed with more sophisticated methods, such as finite element methods (FEM). In a recent work (Buccino et al. 2019), we used FEM simulations and showed that the extracellular probes, especially MEAs, affect the amplitude of the recorded signals. While this finding is definitely interesting for accurately modeling and understanding how the extracellular potential is generated and recorded, it is unclear how it would affect the spike sorting performance. Moreover, when modeling signals on MEAs, we used the method of images (Ness et al. 2015; Buccino et al. 2019), which models the probe as a infinite insulating plane and better describes the recorded potentials for large MEA probes (Buccino et al. 2019).

Secondly, during templates generation, the neuron models were randomly moved around and rotated with physiologically acceptable values (Buccino et al. 2018). In this phase, some dendritic trees might unnaturally cross the probes. We decided to not modify the cell models and allow for this behavior for sake of efficiency of the simulator. The modification of the dendritic trees for each extracellular spike generation would in fact be too computationally intense. However, since the templates generation phase is only run once for each probes, in the future we plan to both to include the probe effect in the simulations and to carefully modify the dendritic positions so that they do not cross the probes’ plane.

Another limitation of the proposed modeling approach is in the replication of bursting behavior. We implemented a simplified bursting modulation that attempts to capture the features recorded from extracellular electrodes by modifying the template amplitude and shape depending on the spiking history. However, more advanced aspects of waveform modulation caused by bursting, including morphology-dependent variation of spike shapes, cannot be modeled with the proposed approach, and their replication requires a full multi-compartment simulation (Hagen et al. 2015). Nevertheless, the suggested simplified model of bursting could be a valuable tool for testing the capability of spike sorters to deal with this phenomenon.

Finally, the current version of MEArec only supports cell models from the Neocortical Microcircuit Portal (Markram et al. 2015; Ramaswamy et al. 2015), which includes models from juvenile rat somatosensory cortex. The same cell model format is also being used to build a full hippocampus model (Migliore et al. 2018) and other brain regions, and therefore the integration of new models should be straightforward. However, we also provide a mechanism to use custom cell models. For example, cell models from the Allen Brain Institute database (Gouwens et al. 2018)^1^, which contains models from mice and humans, can be easily used to simu late templates and recordings, as documented in this notebook: https://github.com/alejoe91/MEArec/blob/master/notebooks/generate_recordings_with_allen_models.ipynb. Other cell models can be used with the same approach.

The use of fully-simulated recordings can raise questions on how well the simulations replicate real extracellular recordings. For example, recordings on freely moving animals present several motion artifacts that are complicated to model and incorporate into simulators. For these reasons, we believe that spike sorting validation cannot be solely limited to simulated recordings. In a recent effort for spike sorting validation, named SpikeForest (Magland et al. 2020), the authors have gathered more than 650 ground-truth recordings belonging to different categories: paired recordings, simulated synthetic recordings (including MEArec-generated datasets), hybrid recordings, and manually sorted data. We think that a systematic benchmark of spike sorting tools will benefit from this larger collection of diverse ground-truth recordings, and in this light, MEArec can provide high-quality simulated datasets to aid this purpose.

In conclusion, we introduced MEArec, which is a Python-based simulation framework for extracellular recordings. Thanks to its speed and controllability, we see MEArec to aid both the development and validation spike sorting algorithms and to help understanding the limitation of current methods, to improve their performance, and to generate new software tools for the hard and still partially unsolved spike sorting problem.

## Information Sharing Statement

The presented software package is available at https://github.com/alejoe91/MEArec and https://github.com/alejoe91/MEAutility(used for probe handling). The packages are also available on pypi: https://pypi.org/project/MEArec/ - https://pypi.org/project/MEAutility/. All the datsets generated for the paper and used to make figures are available on Zenodo at 10.5281/zenodo.3696926, where instruction to generate figures are also provided.

### Electronic supplementary material

Below is the link to the electronic supplementary material.

(PDF 377 KB)

## Appendix: A - Command line interface (CLI)

MEArec implements a command line interface (CLI) to make templates and recordings generation easy to use and to allow for scripting. In order to discover the available commands, the user can use the --help option:

Each available command can be inspected using the --help option:

At installation, MEArec creates a configuration folder (.config/mearec) in which global settings are stored. The default paths to cell models folder, templates and recordings output folders and parameters can be set using the set- commands. By default, these files and folders are located in the configuration folder.

A list of available probes can be found by running the available-probes command.

## Footnotes

1 https://celltypes.brain-map.org/
