# Supplementary material for: MEArec: A Fast and Customizable Testbench Simulator for Ground-truth Extracellular Spiking Activity
Source: Neuroinformatics. 2020 Jul 9;19(1):185–204. doi: 10.1007/s12021-020-09467-7 (PMC7782412; doi:10.1007/s12021-020-09467-7)
Supplement: Supplementary file 1 — (PDF 377 KB) [file 12021_2020_9467_MOESM1_ESM.pdf]

Supplementary methods – MEArec: a fast and customizable testbench simulator for ground-truth extracellular spiking activity

Templates generation

This section explains the templates generation phase of the simulator (Fig. 1A). Table 1 shows the list of parameters involved in this phase, their default values, types, and an explanation of their function.

`MEArec` is compatible with realistic multi-compartment neuronal models from the Neocortex Microcircuit Portal (NMC - [18, 14]). Upon installation, 13 cell models from layer 5 are copied in the package folder. The set of available neurons can be easily extended with more cell models from the NMC portal. In order to do so, the user can download and unzip the cell models in the cell models folder (which can be retrieved using the `mearec default-config` command). In addition to NMC cell models, `MEArec` also has a custom mechanism to load cell models. For example, the custom mechanism can be used to simulate recordings with models from the Allen Institute of Brain Science [8][1], as shown in this notebook.

Intracellular simulation

The neuronal model dynamics is solved using the `NEURON` simulator [5]. The neuron's soma is stimulated with a constant current for a user-defined simulation time (1 secondecond by default - `sim_time` parameter) and the stimulation weight is adjusted (using the `weights` parameter) so that the number of spikes in the simulation period is within a target interval (between 3 and 50 by default - `target_spikes` parameter). The stimulation starts after `delay` ms from the start of the simulation to avoid initialization artifacts. The simulation time step is defined by the parameter `dt` (default is 0.03125 ms, corresponding to 32 kHz). Single spikes are then detected by threshold crossing, aligned, and cropped (using the `cut_out` parameter). The transmembrane currents of all segments are saved to disk, so that the intracellular simulation only needs to be run once for each cell model.

Extracellular simulation

Transmembrane currents generated by the *intracellular simulation* are used to compute extracellular potentials at the electrode locations using `LFPy` [9]. Transmembrane currents are distributed over a line source with the length of its corresponding neural segment. Using the quasi-static approximation [16] and with the assumption of a homogeneous, isotropic, and infinite neural tissue with conductivity $\sigma = 0.3\,\mathrm{S/m}$ [7], the contribution of each compartment i at position $\boldsymbol{r}_i$ with transmembrane current $I_i(t)$ to the electric potential on an electrode at position $\boldsymbol{r}_j$ reads [11, 9, 2]:

$$\phi_i(\boldsymbol{r}_j, t) = \frac{1}{4\pi\sigma} I_i(t) \int \frac{\mathrm{d}r_i}{\|\boldsymbol{r}_j - \boldsymbol{r}_i\|}. \quad (1)$$

[1] `https://celltypes.brain-map.org/`

While the assumption of an infinite milieu holds for small probes, such as microwires and tetrodes, when using larger silicon probes, the use of the method of images (MoI) [15] can yield a better estimate of the extracellular potential [3]. Using MoI, the contribution of a transmembrane current to an electrode at position $\boldsymbol{r}_j$ reads:

$$\phi_i(\boldsymbol{r}_j, t) = \frac{1}{2\pi\sigma} I_i(t) \int \frac{\mathrm{d}r_i}{\|\boldsymbol{r}_j - \boldsymbol{r}_i\|}. \tag{2}$$

The simulated extracellular spike is obtained by summing up the contributions of all compartments. For each recording site, the electric potential can be computed on several points within the electrode area (`ncontacts` parameter - 10 points by default), that are then averaged to model the spatial filtering properties of the electrodes (disk-electrode approximation [13]).

Each cell model, during the *templates generation* phase, is used to generate several spikes (`n` parameter - 50 by default). For each extracellular action potential, the neuron is randomly moved to a position within user-defined boundaries (`xlim`, `ylim`, `zlim` parameters). If the boundary for a specific axis is set to `null`, the limits are computed as the boundary of the probe in that axis plus the `overhang` value (default 30 µm). Moreover, a random rotation of the model can be optionally added (`rot` parameter). The models can be only shifted (norot), rotated along a single axis (xrot, yrot, zrot), rotated with a physiological rotation (physrot), or rotated randomly along all axes (3drot). For further details we refer to [2]. Extracellular spikes are included in the dataset only if their maximum amplitude is greater than a user-defined minimum amplitude (`min_amp` parameter - 30 µV by default). In order to use the *far-neurons* noise model (Fig. 7), the minimum amplitude parameter should be set to 0, so that low amplitude templates are not discarded.

Probe models

Probe models are handled using the `MEAutility` Python package (`https://github.com/alejoe91/MEAutility`), which is automatically installed upon `MEArec` installation. The probe type can be chosen using the `probe` parameter (if not set, a random probe will be selected). `MEAutility` contains a large variety of available probe designs, e.g. commercial Neuronexus probes, Neuropixels [12], and high-density square MEA (Fig. 3), and it also allow users to define new probes using a `yaml` file or a Python dictionary. The probe definition contains information about the number and arrangement of the electrodes, the electrode shape and size (used for spatial filtering), the plane in which electrodes are located, and the probe type (`wire` or `mea`), which tells the simulator whether to use the infinite assumption (Equation 1) or MoI (Equation 2) for the extracellular potential calculation. In order to list the available probes and their information, one can use the `mearec available-probes --info` command.

Drifting templates

When inserting recording probes in the brain, over time there might be relative movement between the probe and the tissue, which causes a so-called drift in the recorded action potentials. In order to incorporate this phenomenon in the simulation of the recordings, drifting templates have to be generated (when the `drifting` parameter is set to true). From an initial random position of the cell model which satisfies the requirements in terms of location (within boundaries) and amplitude (above the detection threshold) a final drifting position is found so that the same conditions are satisfied. Moreover, the user can choose the preferred drifting direction by setting the `drift_xlim`, `drift_ylim`, and `drift_zlim` parameters, which control the drifting limits from the initial position. The minimum and maximum drifting distance can also be set (with the `min_drift` and `max_drift` parameters). When the final position is selected, the cell model is moved along a straight line connecting the initial and final position and the extracellular spike is simulated for `drift_steps` equidistant points (30 points by default) along this line (Fig. 6A).

The templates generation phase can be reproduced by setting the `seed` parameter, which is randomly selected if it is set no `null`.

Parameter	Value	Type	Explanation
**Intracellular simulation settings**			
sim_time	1	float	intracellular simulation time in seconds
target_spikes	[3, 50]	list (int)	min-max number of spikes in sim_time
cut_out	[2, 5]	list (float)	pre-post peak cut_out in ms
dt	0.03125	float	time step in ms (default is 32 kHz)
delay	10	float	stimulation delay in ms
weights	[0.25, 1.75]	list (float)	weights to multiply stimulus amplitude if number of spikes is above (0.25) or below (1.25) target spikes
**Extracellular simulation settings**			
rot	physrot	string	rotation to apply to cell models (norot, xrot, yrot, zrot, physrot, 3drot)
probe	null	string	extracellular probe (if null an available probe is randomly chosen)
ncontacts	10	int	number of contacts per recording site
overhang	30	float	extension in µm beyond MEA boundaries for neuron locations (if corresponding lim is null)
xlim	[10,80]	list (float)	limits (low, high) for neuron locations in the x-axis in µm
ylim	null	list (float)	limits (low, high) for neuron locations in the y-axis in µm
zlim	null	list (float)	limits (low, high) for neuron locations in the z-axis in µm
min_amp	30	float	minimum template amplitude
n	50	int	number of extracellular action potentials per cell model
n_overlap_pairs	null	int	number of spatially overlapping templates
drifting	False	bool	if True, drifting templates are simulated
max_drift	100	float	maximum distance from the initial and final cell position
min_drift	30	float	minimum distance from the initial and final cell position
drift_steps	30	int	number of drift steps
drift_xlim	[-10, 10]	list (float)	limits (low, high) for neuron drift locations in the x-axis
drift_ylim	[-10, 10]	list (float)	limits (low, high) for neuron drift locations in the y-axis
drift_zlim	[20, 80]	list (float)	limits (low, high) for neuron drift locations in the z-axis
seed	null	int	random seed for positions and rotations

Table 1: Templates generation parameter list, values, types, and explanations.

Recordings generation

When a template library is generated, it can be used to generate many recordings, as shown in Fig. 1B. Tables 2 and 3 show the list of parameters involved in the recordings generation phase, their default values, types, and an explanation of their function.

Spike trains generations

In order to obtain the spiking activity, spike trains have to be generated. All the spike train generation parameters can be found in the `spiketrains` section of the recordings parameters.

Spike trains can be generated either as Poisson or Gamma processes (`process` parameter). If the Gamma process is selected, its shape is controlled by the `gamma_shape` parameter (default is 2). The user can decide the number of excitatory (`n_exc`) and inhibitory neurons (`n_inh`) in the recordings. The average and standard deviation of the firing rates of excitatory and inhibitory neurons can be chosen (with the `f_exc`, `f_inh`, `st_exc`, and `st_inh` parameters), as well as the minimum accepted firing rate (`min_rate` - default 0.5 Hz). Alternatively, the user can define the type (E-I) and mean

firing rate of all neurons in the recordings. As Poisson and Gamma processes do not have a minimum inter-spike-interval, spikes violating a refractory period (`ref_per` - 2 ms by default) are removed from the spike trains. Finally, the duration of the spike trains sets the duration of the recordings (`duration` parameter). Spike trains are represented as `neo.SpikeTrain` objects [6].

Spike trains can also be generated externally and provided to the simulator, as shown as shown in this notebook).

Excitatory and inhibitory cell types

The `cell_types` section of the recordings parameters tells the simulator which cell types are excitatory and which are inhibitory. For all cell models in the Neocortical Microcircuit Portal [18], excitatory cells can be pyramidal cells (PC), star pyramidal cells (SP), and stellate cells (SS). The population of inhibitory cells is more diverse and it includes: axon cells (AC), bipolar cells (BP), bitufted cells (BTC), basket cells (BC), Chandelier cells (ChC), double bouquet cells (DBC), Martinotti cells (MC), and neurogliaform cells (NGC) [14]. This substrings are used to identify the cell models belonging to the excitatory and inhibitory group for the template selection process. When using custom models, this dictionary should be overwritten for a correct selection of excitatory and inhibitory templates (as shown in this notebook).

Template selection and pre-processing

After spike trains are generated, templates are selected from the template library and associated with each spike train. The parameters involved in the template selection and pre-processing are in the `templates` section of the recordings parameters.

Templates are chosen based on amplitude, distance, spatial overlap, and cell type. The selection algorithm discards templates with a peak amplitude below and above user-defined threshold (`min_amp` and `max_amp` parameters) and with a distance from already selected neurons below a minimum distance (`min_dist` parameter). Moreover, the user can select specific boundaries in the x-, y-, and z-direction (`xlim`, `ylim`, and `zlim` parameter). If the boundaries are set to `null` (by default), there is no restriction on the neurons' location. Templates are chosen so that the number of excitatory and inhibitory types matches the spike trains' ones. Finally, the user can select the number of spatially overlapping template pairs in the recordings (`n_overlap_pairs` parameter). Two templates A and B are identified as spatially overlapping if the amplitude of template B on the electrode with largest amplitude for template A is above 90% (`overlap_threshold` parameter) of its maximum amplitude, and viceversa.

When templates are selected, they are pre-processed before the convolution operation. First, the templates are padded on both sides (by default extending the templates of 3 ms on each side - `pad_len` parameter) in order to ensure a smooth convolution operation. The template baseline is first removed, then the templates are extended in both directions by linearly interpolating their initial and final values to 0. Finally, this linearly extended template is re-interpolated with a cubic spline.

Next, to model the time variation occurring during sampling, for each template `n_jitter` versions are created (10 by default). Jittering is performed by upsampling the templates (8x by default - `upsample` parameter) and shifting them randomly in time within a sampling period, before downsampling them back to the original sampling frequency.

Recordings construction

In the `recordings` section of the recordings parameters, the user can set several parameters for the recordings generation. If not specified, the sampling frequency of the recordings (`fs` parameter) is the same as the generated templates (32 kHz by default), but the user can choose a different sampling rate. In this case the templates are resampled using a polyphase filter. If the `overlap` parameter is set to true, each spike is annotated as `NO` (no overlap), `TO` (temporal overlap), or `STO` (spatio-temporal overlap). If the `extract_waveforms` parameter is set to true, after the recordings generation the

waveforms are extracted from the recordings and loaded to the spike train objects. The simulation can also be performed in temporal chunks by setting the `chunk_duration` parameter (20 s by default). Chunking is used to reduce the amount of RAM required by the simulation. Different chunks can also be processed in parallel by providing an `n_jobs` (>1) argument when launching the simulation.

Overlapping spikes and spatio-temporal synchrony. Spatio-temporal overlapping of spikes can make spike sorting very challenging [17, 19]. In order to control how spike sorting is affected by the rate of overlapping spikes, `MEArec` enables users to modify the spike trains in order to introduce a controlled amount of spatio-temporal overlapping synchrony (Fig. 5).

If the synchrony rate is set (`sync_rate` parameter), the spike trains of spatially overlapping templates are modified to reach the desired synchrony rate. If the chosen synchrony rate is lower than the initial rate, spatio-temporal overlapping spikes are randomly removed from the spike trains. Conversely, when the chosen synchrony rate is greater than the initial rate, additional spikes that do not violate the refractory period are randomly added to the corresponding spike trains until the desired rate is reached. The additive spikes are jittered randomly within a user-defined interval (`sync_jitt` - default ± 1 *ms*).

Modulated convolution. Pre-processed templates and spike trains are combined with a customized (modulated) convolution. For each spike event of a spike train, a randomized jittered version of the corresponding template is selcted, to reproduce variations due to the finite sampling rate. In order to mimic the variability of spikes in experimental data and computational models [1, 10], the convolution between spike trains and templates is modulated, i.e., the template corresponding to each spike can be modified both in amplitude and in shape (Fig. 4).

There are three types of amplitude `modulation` available: 1) *none* (no modulation), 2) *template*, 3) *electrode* modulation (default). On top of amplitude modulation, when `modulation` is not *none*, shape modulation can be used by setting the `shape_mod` parameter to true.

Amplitude modulation. The amplitude modulation consists of scaling the amplitude of each spike event with a modulation value. When the *template* modulation is selected, the modulation value is the same for all the electrodes. When the *electrode* modulation is used, each electrode has a slightly different modulation value. For the *template* and *electrode* modulation types, if the `bursting` parameter is set to false, the modulation value is a random value drawn from a normal distribution $\mathcal{N}(1, \texttt{sdrand}^2)$, (where `sdrand` is 0.05 by default). As the distribution has mean equal 1, the average amplitude of the resulting modulated spikes is the same as the original template. When the `bursting` parameter is set to true, the modulation values are computed to reproduce the amplitude scaling due to bursting behavior (see Fig. 4A). The user can choose how many units will be affected by bursting (`n_bursting` parameter). Consecutive spikes occurring within a user-defined bursting period (`max_burst_duration` parameter - default 100 ms) are scaled with a sub-linear function (up to a maximum number of consecutive spikes `n_burst_spikes` - 10 by default). The amplitude scaling for the *i*-th consecutive spike within a bursting event is computed as:

$$mod_i = \left(\frac{avg\_isi_{0-i}}{c \cdot max\_burst\_duration}\right)^{exp\_decay}$$

where avg_isi_{0-i} is the average inter-spike-interval (ISI) from the first bursting spike to the current spike in the bursting event, c is the number of consecutive spikes encountered up to spike i, `max_burst_duration` is the maximum bursting period (default 100 ms), and `exp_decay` is the exponent (0.1 by default). Additionally, the ISI-dependent modulation value is scaled a by a random value drawn from a normal distribution at the template level (*template* modulation) or electrode level (*electrode* modulation).

Shape modulation. When `shape_mod` is set to true, spikes are also modulated in shape. Shape modulation consists of stretching the template depending on its modulation value (the same modulation value is used both for amplitude and shape modulation). The stretch is achieved in the following way:

first, the template time axis is centered to the template peak and scaled so that its length is equal to 1 – we will refer to this centered and normalized time axis as x_c; second, x_c is multiplied by the `shape_stretch` parameter, which controls the amount of stretch – we will refer to this transformed time axis as x_t; then, a stretch factor s is computed for the entire template (the same factor is computed for all electrodes) as the average modulation value of all electrodes (if *electrode* modulation is used); if the stretch factor is less than 1, x_t is projected on a sigmoid function:

$$x_s = \frac{1}{\left[1 + \exp^{-(1-s)\cdot x_t}\right]} - 0.5$$

x_s is re-scaled so that its length is `shape_stretch`, and it is now a non-linear stretched time axis. The template is interpolated on x_s with a cubic spline and transformed back to a linear time axis x_r and scaled in amplitude using the modulation value. Fig. S1 shows the different axes involved in shape modulation, and examples of the template transformation at different stages for two modulation values (0.9 and 0.7).

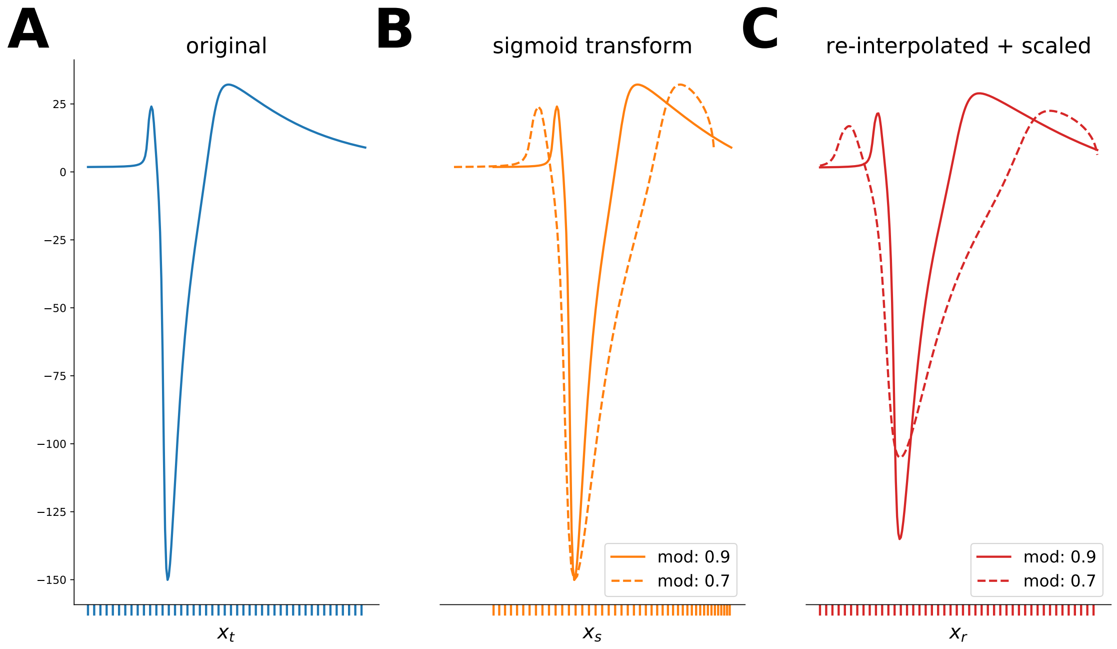

Figure S1: Shape modulation. (A) Original template on a the linear axis x_t. (B) Templates after projection on the sigmoid-transformed axis x_s (solid: modulation value=0.9, dashed: modulation value=0.7). Note that different modulation values, and in turn different s values, make the x_s range vary. (C) Templares after re-interpolation on the linear x_r and amplitude scaling by the modulation value (solid: modulation value=0.9, dashed: modulation value=0.7).

Noise models and post-processing Additive noise is superimposed to the signals after the modulated convolution is finished. There are three types of noise models that can be set using the `noise_mode` parameter: *uncorrelated*, *distance-correlated*, and *far-neurons*. The uncorrelated noise model is an additive Gaussian noise with a user-defined standard deviation (`noise_level` parameter - 10 µV by default). The distance-correlated mode generates a multivariate normal noise with a covariance matrix dependent on the distance between electrodes. The covariance between electrode i and j is defined as $c_{ij} = {}^{d_h}/{}_{2 \cdot d_{ij}}$, where d_{ij} is the distance between the electrodes and d_h is the distance

Parameter	Value	Type	Explanation
**Spike trains**			
n_exc	7	int	number of excitatory cells
n_inh	3	int	number of inhibitory cells
f_exc	5	float	average firing rate of excitatory cells in Hz
f_inh	15	float	average firing rate of inhibitory cells in Hz
st_exc	1	float	firing rate standard deviation of excitatory cells in Hz
st_inh	3	float	firing rate standard deviation of inhibitory cells in Hz
min_rate	0.5	float	minimum firing rate in Hz
ref_per	2	float	refractory period in ms
process	poisson	string	process for spike train simulation (poisson-gamma)
gamma_shape	2	float	gamma shape (for gamma process)
duration	10	float	duration in seconds
**Cell types**			
excitatory	['PC', 'SS', 'SP']	list (string)	Excitatory cell types
inhibitory	['AC', 'BP', 'BC', 'BTC', 'ChC', 'DBC', 'MC', 'NGC']	list (string)	Inhibitory cell types
**Templates**			
min_dist	25	float	minimum distance between neurons
min_amp	50	float	minimum spike amplitude in µV
max_amp	500	float	maximum spike amplitude in µV
xlim	null	list (float)	limits for neuron x in µm (min, max)
ylim	null	list (float)	limits for neuron y in µm (min, max)
zlim	null	list (float)	limits for neuron z in µm (min, max)
overlap_threshold	0.9	float	threshold to consider two templates spatially overlapping
n_jitters	10	int	number of temporal jittered copies for each template
upsample	8	int	upsampling factor to extract jittered copies
pad_len	[3, 3]	list (float)	padding of templates in ms

Table 2: Recordings generation parameter list, values, types, and explanations.

at which the covariance is 0.5 (`noise_half_distance` parameter - 30 µm by default). Finally, the *far-neurons* model generates noise as the activity of many neurons (`far_neurons_n` parameter - 300 by default) with small amplitudes (below `far_neurons_max_amp` - 10 µV by default). The population of distant neurons has an excitatory/inhibitory ratio of `far_neurons_exc_inh_ratio` (default 0.8). A random noise floor with a standard deviation of `far_neurons_noise_floor` (default 0.5) times the standard deviation of the distant neurons' spiking activity is added, in agreement with experimental data [4].

Uncorrelated and distance-correlated noise types can also be modulated in frequency to match the spectrum observed in experimental data [4, 9]. Extracellular spiking activity exhibit a peak in frequency at around 300 Hz, a $1/f$ spectrum, and a random noise floor. Noise can be *colored* (when the `noise_color` parameter is true) with a second order infinite impulse response (IIR) peak filter and an additional gaussian noise floor. The frequency peak, quality factor, and weight of the random noise floor can be set with the `color_peak`, `color_q`, and `color_noise_floor` parameters. Note that with distance-correlated noise the correlation is slightly reduced by the color filter, as a random noise floor is added.

Optionally, the signals can be filtered (by setting the `filter` to true) with an high-pass or band-pass Butterworth filter of order `filter_order` (3 by default) and cutoff frequencies of `filter_cutoff` ([300, 6000] Hz by default).

Drifting recordings When the `drifting` parameter is set to true, drifting recordings are generated. The template library must have been generated with the `drifting` mode as well. The user can decide the number of drifting units (`n_drifting` parameter). If `n_drifting` is `null`, all units will be drifting.

The generation of drifting recordings is only different in the template selection and modulated convolution steps. In the template selection, in addition to the selection rules based on template amplitude, inter-neuron distance, and spatial overlap, templates are selected if the angle between the

Parameter	Value	Type	Explanation
**Recordings**			
fs	null	int	sampling frequency in Hz<br>(if null it is computed form the templates)
dtype	float32	dtype	dtype of generated recordings
overlap	False	bool	if True, temporal and spatial overlap are computed<br>for each spike (it may be time consuming)
extract_waveforms	False	bool	if True, waveforms are extracted from recordings
sync_rate	null	float	synchrony rate ([0-1]) for spike trains<br>of spatially overlapping templates
sync_jitt	1	float	jitter in ms for added synchronous spikes
modulation	electrode	string	type of modulation [none | template | electrode]<br>none - no modulation<br>template - each spike instance is modulated with the<br>same value on each electrode<br>electrode - each electrode is modulated separately
sdrand	0.05	float	standard deviation of Gaussian modulation
bursting	False	bool	if True, spikes are modulated in amplitude depending<br>on the ISI
exp_decay	0.1	float	(bursting) experimental decay in<br>amplitude between consecutive spikes
n_burst_spikes	10	int	(bursting) max number of 'bursting' consecutive spikes
max_burst_duration	100	float	(bursting) duration in ms of maximum burst modulation
shape_mod	False	bool	if True waveforms are stretched in shape with a sigmoid<br>transform depending on their modulation value
shape_stretch	30	float	amount of stretch for shape modulation
n_bursting	null	int	number of bursting units. If null all units are bursting
chunk_duration	20	float	chunk duration (if running into MemoryError)
noise_level	10	float	noise standard deviation in uV
noise_mode	uncorrelated	string	[uncorrelated | distance-correlated | far-neurons]
noise_color	False	bool	if True noise is colored resembling experimental noise
noise_half_distance	30	float	(distance-correlated) distance between<br>electrodes in µm for which correlation is 0.5
far_neurons_n	300	int	(far-neurons) number of far neurons to be simulated
far_neurons_max_amp	10	float	(far-neurons) maximum amplitude of far neurons
far_neurons_noise_floor	0.5	float	(far-neurons) percent of additive random noise
far_neurons_exc_inh_ratio	0.8	float	(far-neurons) excitatory / inhibitory noisy neurons ratio [0-1]
color_peak	300	float	(color) peak / cutoff frequency of resonating filter in Hz
color_q	2	int	(color) quality factor of resonating filter
color_noise_floor	0.5	float	(color) percent of additive random noise
filter	True	bool	if True recordings are filtered
filter_cutoff	[300, 6000]	float/list	filter cutoff frequencies in Hz
filter_order	3	int	filter order
drifting	False	bool	if True drifting recordings are simulated
n_drifting	null	int	number of drifting units. If null all units are drifing
preferred_dir	[0, 0, 1]	list	preferred drifting direction<br>([0, 0, 1] is positive z, direction)
angle_tol	15	float	tolerance for direction in degrees
slow_drift_velocity	5	float	slow drift velocity in µm/min
fast_drift_period	float	20	period between fast drift events in s
fast_drift_max_jump	float	20	maximum amplitude jump in µV for fast drifts
fast_drift_min_jump	float	5	minimum amplitude jump in µV for fast drifts
t_start_drift	0	float	time in seconds after which drift starts
**Seeds**			
spiketrains	int	null	seed for spike trains generation
templates	int	null	seed for template selection
convolution	int	null	seed for convolution operation
noise	int	null	seed for noise generation

Table 3: (Continued) Recordings generation parameter list, values, types, and explanations.

drifting direction (computed as the vector connecting the final and initial position) and a user-defined preferred direction (`preferred_dir` parameter - [0, 0, 1] by default) is within an angle tolerance (`angle_tol` parameter - 15° by default).

There are three types of drift modes available (`drift_mode` parameter): *slow*, *fast*, and *slow+fast*. The different modalities vary in terms of how the drifting template is selected for each spike during the modulated convolution.

For *slow* drifts, a new position is calculated moving from the initial position along the drifting direction with a velocity of `slow_drift_velocity` (default 5 µm/*min*). If a boundary position is

reached (initial or final positions), the drift direction is reversed.

For *fast* drifts, the user can set the frequency at which fast drift events occur (every `fast_drift_period` s). When a fast drift event happens, a new template position is selected randomly among the drifting templates for each drifting neuron. The amount of *jump* is controlled by the `fast_drift_min_jump` and `fast_drift_max_jump` in the following way. Let us call T_{old} the drifting template before the drift event, and T_{rand} the randomly selected template, which is a candidate for the new template after the fast drift event. T_{rand} is accepted as the new template only if the difference in amplitude $diff_{amp}$ between T_{old} and T_{rand} on the channel in which T_{old} has the largest peak is `fast_drift_min_jump` $<$ $diff_{amp}$ $<$ `fast_drift_max_jump`. This is to ensure that fast drifts are not too abrupt.

Finally, when the *slow+fast* mode is selected, the two previously described modes are combined. In all cases, the user can decide to start the drift `t_start_drift` seconds after the start of the recordings.

Reproducibility and seeds

In order to ensure full reproducibility of the recordings, the `seeds` section of the recordings parameters enables users to set several random seeds involved in the simulations. The `spiketrains` seed controls the random generation of spike trains, the `templates` seed the selection of templates from the template library, the `convolution` seed is for all the processes involved in the convolution phase (modulation, jittering, drifting), and the `noise` seed controls the randomness in the noise generation. If any of the seed is not set, a random seed is generated and saved in the recording output (in the `info` dictionary), so that the recordings could be reproduced in the future.

References

[1] B. D. Allen, C. Moore-Kochlacs, J. G. Bernstein, J. Kinney, J. Scholvin, L. Seoane, C. Chronopoulos, C. Lamantia, S. B. Kodandaramaiah, M. Tegmark, et al. Automated in vivo patch clamp evaluation of extracellular multielectrode array spike recording capability. *Journal of neurophysiology*, 2018.

[2] A. P. Buccino, M. Kordovan, T. V. Ness, B. Merkt, P. D. Häfliger, M. Fyhn, G. Cauwenberghs, S. Rotter, and G. T. Einevoll. Combining biophysical modeling and deep learning for multielectrode array neuron localization and classification. *Journal of neurophysiology*, 2018.

[3] A. P. Buccino, M. Kuchta, K. H. Jæger, T. V. Ness, P. Berthet, K. A. Mardal, G. Cauwenberghs, and A. Tveito. How does the presence of neural probes affect extracellular potentials? *Journal of neural engineering*, 2019.

[4] L. A. Camuñas-Mesa and R. Q. Quiroga. A detailed and fast model of extracellular recordings. *Neural computation*, 25(5):1191–1212, 2013.

[5] N. T. Carnevale and M. L. Hines. *The NEURON book.* Cambridge University Press, 2006.

[6] S. Garcia, D. Guarino, F. Jaillet, T. R. Jennings, R. Pröpper, P. L. Rautenberg, C. Rodgers, A. Sobolev, T. Wachtler, P. Yger, et al. Neo: an object model for handling electrophysiology data in multiple formats. *Frontiers in neuroinformatics*, 8:10, 2014.

[7] T. Goto, R. Hatanaka, T. Ogawa, A. Sumiyoshi, J. Riera, and R. Kawashima. An evaluation of the conductivity profile in the somatosensory barrel cortex of wistar rats. *J Neurophysiol*, 104(6):3388–3412, 2010.

[8] N. W. Gouwens et al. Systematic generation of biophysically detailed models for diverse cortical neuron types. *Nature communications*, 9(1):710, 2018.

[9] E. Hagen, S. Næss, T. V. Ness, and G. T. Einevoll. Multimodal modeling of neural network activity: Computing lfp, ecog, eeg, and meg signals with lfpy 2.0. *Frontiers in neuroinformatics*, 12, 2018.

[10] E. Hagen, T. V. Ness, A. Khosrowshahi, C. Sørensen, M. Fyhn, T. Hafting, F. Franke, and G. T. Einevoll. Visapy: a python tool for biophysics-based generation of virtual spiking activity for evaluation of spike-sorting algorithms. *Journal of neuroscience methods*, 245:182–204, 2015.

[11] G. R. Holt and C. Koch. Electrical interactions via the extracellular potential near cell bodies. *J Comput Neurosci*, 6(2):169–184, 1999.

[12] J. J. Jun, N. A. Steinmetz, J. H. Siegle, D. J. Denman, M. Bauza, B. Barbarits, A. K. Lee, C. A. Anastassiou, A. Andrei, Ç. Aydın, et al. Fully integrated silicon probes for high-density recording of neural activity. *Nature*, 551(7679):232, 2017.

[13] H. Lindén, E. Hagen, S. Leski, et al. LFPy: a tool for biophysical simulation of extracellular potentials generated by detailed model neurons. *Frontiers in Neuroinformatics*, 7:41, 2014.

[14] H. Markram, E. Muller, S. Ramaswamy, et al. Reconstruction and simulation of neocortical microcircuitry. *Cell*, 163(2):456–492, 2015.

[15] T. V. Ness, C. Chintaluri, J. Potworowski, S. Łęski, H. Głąbska, D. K. Wójcik, and G. T. Einevoll. Modelling and analysis of electrical potentials recorded in microelectrode arrays (meas). *Neuroinformatics*, 13(4):403–426, 2015.

[16] P. L. Nunez and R. Srinivasan. *Electric fields of the brain: the neurophysics of EEG*. Oxford University Press, USA, 2006.

[17] M. Pachitariu, N. A. Steinmetz, S. N. Kadir, et al. Fast and accurate spike sorting of high-channel count probes with kilosort. In *Advances in Neural Information Processing Systems*, pages 4448–4456, 2016.

[18] S. Ramaswamy, J. Courcol, M. Abdellah, et al. The neocortical microcircuit collaboration portal: a resource for rat somatosensory cortex. *Front Neural Circuits*, 9, 2015.

[19] P. Yger, G. L. Spampinato, E. Esposito, B. Lefebvre, S. Deny, C. Gardella, M. Stimberg, F. Jetter, G. Zeck, S. Picaud, et al. A spike sorting toolbox for up to thousands of electrodes validated with ground truth recordings in vitro and in vivo. *Elife*, 7:e34518, 2018.
